# Cardiac System during the Aging Process

**DOI:** 10.14336/AD.2023.0115

**Published:** 2023-08-01

**Authors:** Ana Sofia Fernandes Ribeiro, Blanca Egea Zerolo, Fidel López-Espuela, Raúl Sánchez, Vítor S Fernandes

**Affiliations:** ^1^Escuela de Enfermería y Fisioterapia San Juan de Dios. Universidad Pontificia Comillas, Madrid, Spain.; ^2^Metabolic Bone Diseases Research Group, Nursing and Occupational Therapy College, University of Extremadura, Caceres, Spain.; ^3^Unidad de Cardiopatías Congénitas, Hospital Universitario La Paz, Madrid, Spain.; ^4^Departamento de Fisiología, Facultad de Farmacia, Universidad Complutense de Madrid, Madrid, Spain.

**Keywords:** Cardiovascular system, aging, fibrosis, amyloidosis, mitochondrial dysfunction genetics, epigenetics, exercise

## Abstract

The aging process is accompanied by a continuous decline of the cardiac system, disrupting the homeostatic regulation of cells, organs, and systems. Aging increases the prevalence of cardiovascular diseases, thus heart failure and mortality. Understanding the cardiac aging process is of pivotal importance once it allows us to design strategies to prevent age-related cardiac events and increasing the quality of live in the elderly. In this review we provide an overview of the cardiac aging process focus on the following topics: cardiac structural and functional modifications; cellular mechanisms of cardiac dysfunction in the aging; genetics and epigenetics in the development of cardiac diseases; and aging heart and response to the exercise.

## 1. Age-dependent cardiac structural and functional modifications

### 1.1 Ventricular structural modifications

**Figure 1.**
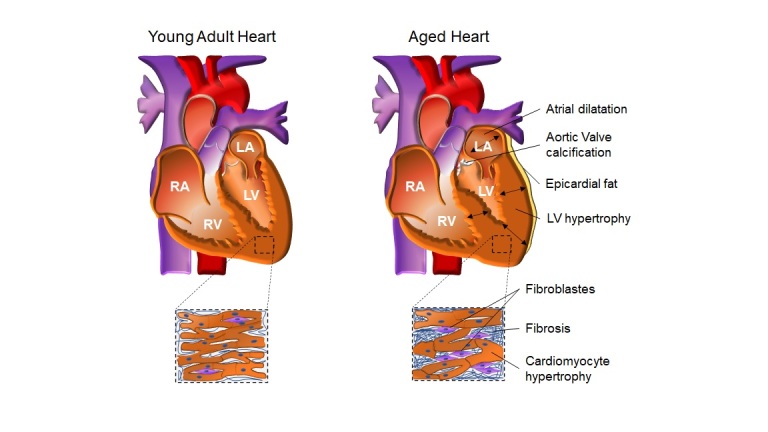
**Age-dependent cardiac structural modifications**. The aged heart shows several structural changes when compared with younger adults. These cardiac modifications include epicardial fat deposition, aortic valve calcification, atrial hypertrophy, dilatation, and concentric left ventricle hypertrophy. At the cellular level, cardiomyocytes apoptosis is observed, and the remaining cardiomyocytes are under hypertrophy. The fibroblasts cells and fibrosis augments with aging. These age-dependent structural modifications may be affected by factors including gender (modified from ref. [8]).

**Figure 2.**
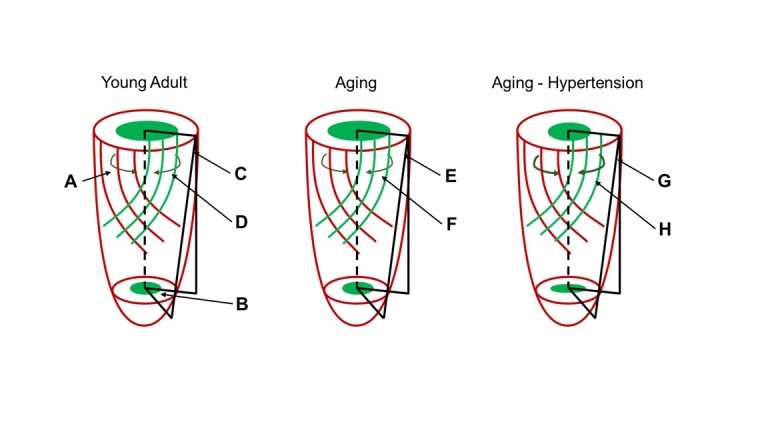
**Epicardial torsion and endocardial circumferential shortening and relationship to subepicardial and subendocardial fiber orientations**. Epicardium is represented in red and endocardium in green. Younger adults: (A) the heart presents oblique subepicardial fibers that lead to a rotation of the apex regarding the base (B) and a clockwise direction. This rotation is quantified in terms of circumferential-longitudinal angle (C). Epicardial torsion has a larger radius, giving it a mechanical advantage over the subendocardium, driving subendocardial bundles to narrow in a direction nearly 90º away from the subendocardial bundle’s orientation (circumferential plane) (D). The torsion to shortening ratio (TSR) quantifies the subepicardial to subendocardial interaction. Augmented values of TSR indicate reduced subepicardial effect over the subendocardium. Aging: there is an increase in the rotational angle E and torsional angle F. Endocardial circumferential shortening does not change compared with young adults, thus TSR increases, suggesting a reduced interaction between subepicardium and subendocardium. Aging - hypertension: there are no changes in the rotational and torsional angles when compared with young adults (G), even though the endocardial circumferential shortening is increased (thick dark green arrows, shortening H), indicating augmented interaction between the subepicardium and subendocardium and hence, the TSR is reduced (modified from ref. [10]).

The most observed structural cardiac modification accompanying aging mainly affects the left ventricle (LV) wall [1,2]. Even without hypertension or another cause of augmented cardiac afterload, during the aging process is observed a moderate increase of the LV wall [3-5], that leads to concentric hypertrophy (defined by the rise of the LV wall with a reduction of the chamber size) (Fig. 1). The aging process can also affect the intraventricular septum size, a cause of LV outflow obstruction resulting in further augmented LV afterload. Blood pressure has been described to increase with age, contributing to LV hypertrophy. However, neurohormonal and other molecular factors cannot be discarded in the genesis of LV hypertrophy development [6]. Indeed, women experience a greater age-related concentric hypertrophy [6] and a fast increase of LV thickness than men [7]. In addition, an increase of the LV thickness with aging in both genders has been demonstrated to be much more pronounced in those individuals with an increased burden of cardiovascular risk factors such as higher body mass index and diabetes besides the blood pressure [5]. The LV mass-to-volume ratio increase in both genders, although whether LV mass increases significantly still controversial [8]. The LV torsion and strain have been described to be altered with aging. The peak LV torsion seems to increase, whereas endocardial and whole wall circumferential strain decrease during cardiac aging, indicating a reduced capability of the subepicardium to influence the subendocardial shortening [9,10]. The epicardial circumferential shortening decreases in individuals with hypertension while endocardial circumferential shortening increases [11]. Moreover, longitudinal shortening diminishes, suggesting an augmented subendocardial function with an increment of the subepicardium and subendocardium interactions (Fig. 2) [10].

### 1.2 Atrial structural modifications

The significant age-related atrial modification occurs in the left atrium (LA) with a progressive increase of the chamber diameter (Fig. 1) in both genders [12-14]. The diastolic filling pressure increases during the cardiac aging process due to a slow early LV filling, leading to atrial dilatation and hypertrophy [15]. In consequence, the contractile force of the atria augments and promotes the late diastolic filling as a compensatory mechanism for the reduced early diastolic filling. Thus, in older adults, the atria contribute more to the ventricular filling than they play in younger adults. Even these changes have been ascribed, in part to the age-associated remodeling of the cardiac tissue composition [17]; more recent studies have shown a strong correlation between LA enlargement and a burden of risk factors that go along with aging [12,18]. Thus, pathophysiological processes such as atrial fibrillation, an age-related disease, attenuate the atria's ability to contract appropriately, contributing to the LA enlargement and reducing diastolic volume in the elderly [15]. Evidence points out a greater incidence of atrial fibrillation in men, while women seem to have worse outcomes for thromboembolic events than males. However, whether gender influences age-related LA enlargement is not yet clear. [8]. The age-related LA volume index (determined by correcting the LA volume to the body surface area) did not differ between sex [19,20]. Individuals with low, intermediate, and high-risk factors, including adiposity, high blood pressure, or diabetes, were shown to have increased the LA diameter in a proportion like 20 years of aging [19]. Hypertensive disease in animal models is aggravated by a high-fat diet and leads to LA enlargement that is also correlated with aging [21]. The treatment with antihypertensive drugs enlarges the LA diameter at baseline. Those receiving antihypertensive treatment present a more pronounced LA diameter during the aging process, suggesting a crucial role in determining the LA remodeling at baseline and aging [12,22].

### 1.3 Cardiac cellular modification

Age-related structural changes occurring at cellular levels are often observed in endothelial cells, cardiomyocytes, pericardial cells, cells of the conduction system, and other cells such as fibroblasts. During the aging process, endocardial cells become thicker with some opaque areas, which seem to be more noted in the LA [23]. Moreover, endocardial thickening is observed in the borderline of the valves, where the degree of structural modifications is related to age-dependent valve thickening [24]. The most common structural modification of the epicardium includes the adipocyte cells deposition along the right and LV with areas of fibrosis [25-27]. Other age-dependent modifications include a decrease in the total number of cardiomyocytes due to autophagy and/or apoptosis [28,29], which is more prevalent in men than women [17]. The remaining cardiomyocytes undergo a hypertrophic state (concentric and eccentric) as a compensatory mechanism for the loss of cardiomyocytes. Cardiomyocyte hypertrophy and age-dependent peripheral vascular stiffening may contribute to ventricular wall thickness [30]. Furthermore, cardiac aging is characterized by cardiac fibroblast proliferation, collagen decompensation, and interstitial fibrosis in the atria and ventricles [31,32]. In aged subjects, there is an increase in collagen type I (bigger tensile strength), whereas collagen type II (more distensibility) decreases [33]. This decompensation could be related to the LV wall stiffening and impaired ventricular mechanical function [33-35]. Moreover, changes in the extracellular matrix surrounding cardiomyocytes contribute to impairments in cell-to-cell electrical coupling, which may result in arrhythmias [35,36]. The cellular function of the conduction system is also affected by collagen deposition, fat tissue, amyloid, and fibrosis, contributing to an arrhythmogenic state. A significant reduction in the number of pacemaker cells in the sinoatrial node, atrioventricular node, and the rest of the conduction system also accompanies these age-dependent modifications [37].

### 1.4 Diastolic and systolic function

The aging process leads to cardiovascular function decline, including ventricular diastolic function [38]. In the elderly, there are slower myocardial contractions and less complete ventricular relaxation compared with younger adult individuals [39,40]. This impaired myocardial contractility is may due to modification in the Ca^2+^ cycling/handling [39]. Different studies have reported that prolonged diastolic relaxation is related to the increased oxidative stress in the senescent myocardium, leading to oxidative damage of the sarcoplasmic reticulum Ca^2+^-ATPase (SERCA) pump and thus to the Ca^2+^-machinery dysregulation [41,42].

In addition, ventricular-arterial stiffening in the senescence myocardium has been described as significant contributor to the development of age-related diastolic dysfunction [39,43]. Cardiovascular stiffening can be ascribed to changes in the extracellular matrix and factors intrinsic to the myocytes. Alterations in the extracellular matrix involve myofibroblast synthesis, collagen degradation impairments, and a shift in the balance between matrix metalloproteinases (downregulation) and tissue inhibitors of metalloproteinases (upregulation) [28]. Cardiomyocytes are long lifespan cells and thus, can accumulate damage resulting from oxidative stress, mitochondrial dysfunction, and lipofuscin. Severe damage leads to cell apoptosis or autophagy. The remaining cells undergo molecular adaptations that promote cell hypertrophy to fill the functional demands of the aging heart [44]. Hence, myocardial wall and extracellular matrix remodeling contribute to diastolic dysfunction in the elderly. Interestingly, these age-related factors are also associated with heart failure with preserved ejection fraction in elderly patients, mainly in women. Indeed, the incidence of heart failure triples with each decade between 65 and 85 years in women, whereas in men, the incidence increases double [45]. The LV concentric remodeling, along with worst diastolic function observed in aged women than men, may underlie the higher incidence of heart failure, mainly with preserved ejection fraction, observed in women [46]. While studies have shown that diastolic function declines with aging, the systolic function (characterized by LV ejection fraction) at rest is usually preserved in aging. Under high-demand situations such as exercise, the ability to augment the ventricular contractility is affected in older adults [47]. Evidence from more sensitive methods to measure systolic function indicate a gradual age-related decline of LV longitudinal strain and strain rate [48] and peak systolic tissue velocity [49]. The systolic phase involves a complex interplay between myocardial fiber that leads to longitudinal shortening and circumferential radial torsion to execute the ejection. During normal aging it is observed a prolonged isovolumic time (reflects the global efficiency of the ventricle) and a Tei index (the sum of the two isovolumic times, relative to ejection time) suggesting a worsening LV dyssynchrony [50]. Moreover, LV twist and torsion have been demonstrated to increase with age [51,52]. The increase of LV torsion and twist results as a compensatory mechanism to maintain stroke volume and cardiac output observed in the age-related decrease of the subendocardial function. Gender did not influence LV rotation and twist [51].

### 1.5 Cardiac autonomic nervous and conduction system dysfunction

The cardiovascular function is controlled by intrinsic and extrinsic mechanisms involving, the last, the autonomous nervous system (ANS) and endocrine system [53]. Normal aging is linked to a decline in the cardiac autonomic nervous function due to several modifications on the ANS [54-56]. These modifications involve impaired central integrations, baroreceptors output, afferent and efferent neuronal conduction, and a decrease of the sinoatrial node response. In addition to the vascular stiffening, the neuronal function largely regulates the cardiovagal gain observed in older individual [57]. Moreover, a diminished neuronal transduction in aging is consistent with impaired modifications of the autonomic integration and a decreased muscarinic receptors function and density [58]. The age-related changes in the baroreceptors leads to an increase of the plasmatic concentrations of catecholamine and thus, elevated muscle sympathetic nerve activity which contributes to the impaired endothelial function and arterial stiffness [59]. In addition, in older adults, beta-and alpha-adrenergic regulation of cardiovascular function, thermoregulation and the renin angiotensin system activity decrease, important factors that regulate blood pressure, blood flow and heart rate. The incidence of arrythmias increases during aging. Indeed, the heart rate variability (HRV), that integrate all these complex interactions, is an indicator of arrhythmic complications and a predictor of sudden death [60]. During aging, there is an imbalance between parasympathetic and sympathetic nervous activity that are responsible for the decrease of HRV [61]. In turn, this imbalance promotes the occurrence of cardiovascular events [62].

**Figure 3.**
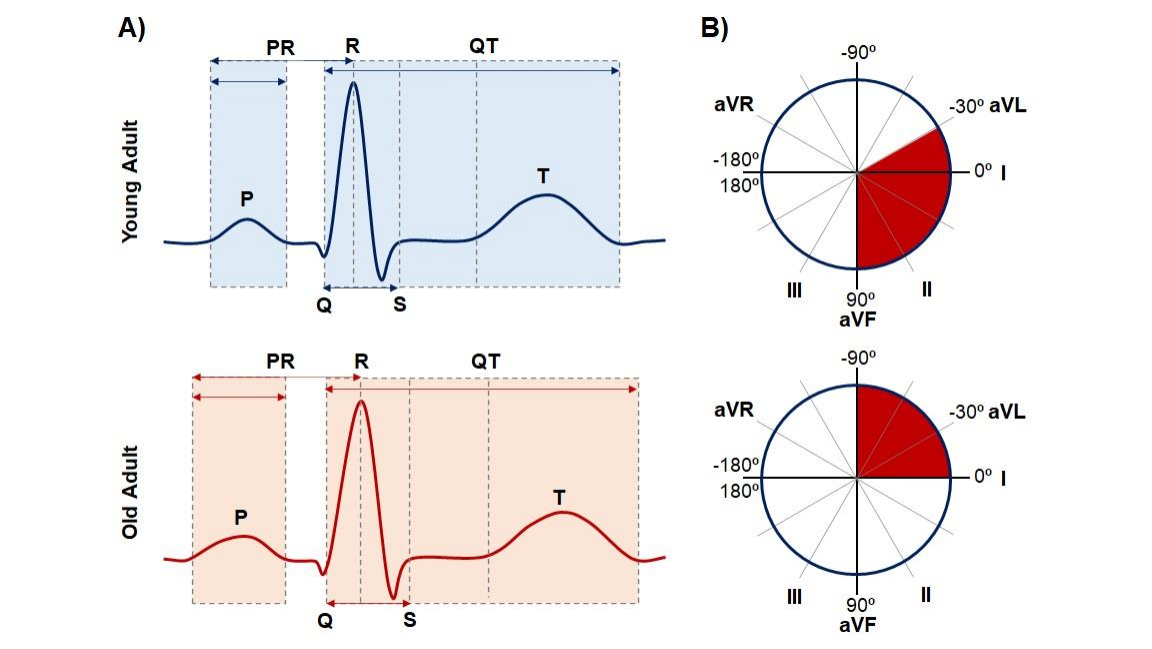
**Normal age-associated changes in ECG measurements**. A) Young adult vs old adult. The P wave represents auricular depolarization. P wave duration can exhibit a minor increase in old adult individuals. The PR interval, representing atrioventricular conduction, increases from 159 ms (individuals averaging 25-35 yrs) to 172 ms (old individual). The QT represents the time taken for ventricular depolarization and repolarization. The QT interval slightly increases with aging heart. Other findings that may became more prevalent with aging such as increased QRS voltage, Q waves, QT interval prolongation, and ST-T-wave anomalies, are usually linked to an increase of cardiovascular risk. B) Cardiac axis, young adult, vs old adult. With aging, individual exhibits a leftward shift of the QRS axis (Based on [16]).

The cardiac conduction system is also affected by aging [37]. It has been reported that with increase aging, there is a decline in the number of pacemaker cells and ion channels expression in the sinoatrial node. This age-related decline of pacemaker cells and ion channels are linked to reduced conduction system automaticity and lower HR [63,64]. Factors such as adipose tissue deposition, collagen and amyloid also play an important role in the cardiac conduction system modifications. All these structural changes, from the production to the conduction of the electrical stimulus, affects the electric activity of the myocardium, hence promotes the clinical appearance of bradyarrhythmia [63-65]. Moreover, electrocardiogram (ECG) of old individuals without evidences of cardiovascular disease may exhibits a mild prolongation of the PR interval, a leftward shift of the QRS axis [16] and a slightly prolonged QRS complex (Fig. 3), reflecting the decrease of electrical waves propagation throughout myocardium [63,66]. Likewise, it is observed and increase in the prevalence, density, and complexity of ectopic beats, both atrial and ventricular. In general, these ECG modifications have not clinically relevance in healthy older adults, however, when occurs ECG changes such as increased QRS voltage, Q waves, QT interval prolongation, and ST-T-wave anomalies, became more prevalent with aging, they are usually linked to an increase of cardiovascular risk [16]. The propagation of the electrical impulse throughout the myocardium depends on the preserved structure and function of the intercalated discs. The gap junctions are integrating components of the junctional complex that contains clusters of low-resistance ionic channels, formed by connexin hemichannels. In the ventricular myocardium, the connexin hemichannels are mainly formed by connexin 43 (Cx43) [67]. In the aging heart, the expression levels of Cx43 decrease, which affects the cell-to-cell electrical connectivity, thus, slowing the conduction of the depolarization wave and contributing to the age-related arrhythmic process [68].

## 2. Cellular mechanisms of cardiovascular dysfunction in aging

### 2.1 Cardiac fibrosis

The cardiac system comprises cells from several lineages and even most of the cardiac cellular population are cardiomyocytes, also comprises cells such as cardiac fibroblasts, smooth muscle cells, endothelial cells, and cardiac stem cells. During the cardiac aging process is observed a structural remodeling at the cellular levels due to a gradual decline of cellular population and an increase of fibrosis [69,70]. Cardiac fibrosis in aging heart results from the activation of fibrogenic factors and the inhibition of the anti-fibrotic mechanisms, resulting in an excessive deposition of components of extracellular matrix (ECM) [34]. A dysregulation between the synthesis and degradation of collagen and fibronectin, leads to accumulation of fibrous connective tissue and thus, cardiovascular structure disarrangement [71]. The ECM is regulated by matrix metalloproteinases (MMPs) and the tissue inhibitors of metalloproteinases (TIMPs). MMPs are proteinases that play an important role regulating collagen degradation whereas TIMPs, by inhibiting MMPs, decreases the collagen degradation [28]. The regulation of MMPs and TIMPs is maintained by several cytokines, platelet-derived growth factor (PDGF), insulin-like growth factor 1 (IGF-1), epidermal growth factor (EGF), transforming growth factor β (TGFβ), heparin-binding EGF-like growth factor (HB-EGF), and granulocyte-macrophage colony-stimulating factor (GM-CSF) [72-74]. The cardiac-aging is accompanied by a downregulation of MMPs, (MMP-1, MMP-2, MMP-4, and MMP-14) and an upregulation of pro-fibrotic inhibitors (TIMP-1 and TIMP-4) [75], which leads to an impaired balance of the ECM turnover and the instauration of cardiovascular fibrosis [76]. In the other hand, the up mentioned factors are capable to induce fibroblast proliferation and remodeling, a key cellular process in the development of fibrotic tissue [77]. With aging, there is a decrease in the number of cardiomyocytes, due to apoptosis and the remaining cardiomyocytes undergo hypertrophy with increase myocardium fibrosis [78].

Prolonged inflammation has been established as a key factor promoting cardiovascular fibrosis. Even though the underline mechanisms are still not well understood, some reports point cytokines, angiotensin II (Ang-II) and AMP-activated protein kinase (AMPK) as important players in the development of cardiovascular fibrosis.

Transforming growth factor-beta (TGF-β) induces myocyte hypertrophy and cardiac fibrosis. Specifically, TGF-β1 induces collagen synthesis via both SMAD3-dependent mechanisms and involving the mitogen-activated protein kinase cascades. In individuals with heart failure with preserved ejection fraction (HFpEF) has been demonstrated higher levels of endocardial TGF-ẞ1 in association with reduced MMP-1 and elevated TIMP-1 levels which suggest an upregulation of collagen synthesis that favor the appearance of cardiac fibrosis [79].

Galectine-3 (Gal-3), a member of the lectin family, increases with aging. An upregulation of Gal-3 has been demonstrated to promote collagen synthesis, deposition, and fibrosis. Indeed, Gal-3 turns quiescent fibroblasts into active myofibroblasts, and thus, increasing the collagen deposition, and ventricular dysfunction [80] whereas pharmacological or genetic inhibition of Gal-3 attenuates cardiac remodeling and myocardial fibrosis [81]. Finally, an upregulation of Gal-3 by Ang-II [81] and aldosterone [82] is involved in the fibrotic remodeling which underlines the role of renin-angiotensin- aldosterone in cardiovascular fibrogenesis. In fact, Ang-II has been demonstrated that can increase fibroblast activity and proliferation, thus, it augments collagen deposition and leads to cardiac hypertrophy [83,84]. In mouse, the administration of Ang-II increases the expression levels of collagen types I and II in the myocardium, leading to interstitial fibrosis, in a mechanism dependent of monocyte/macrophage chemoattractant protein 1 (MCP-1). MCP-1 KO mice did not exhibit myocardial fibrosis after Ang-II administration, which further suggests the role of Ang-II in the cardiovascular fibrogenesis [84].

The AMP-activated protein kinase (AMPK) is a member of the serine/threonine kinase family highly expressed in cardiac tissue. Different studies have demonstrated that AMPK levels and activity in cardiomyocytes and myofibroblasts decrease with aging [85-87]. AMPK-KO mice has increased collagen deposition and cardiac fibrosis leading to cardiovascular dysfunction [88]. In the other hand, it has been reported that AMPK has antifibrotic effects on cardiac cells [85,89]. In fact, AMPK plays a cardioprotective effect in myocardial ischemia, diabetic cardiac injury, and angiogenesis through reparative fibrosis [85,89,90]. AMPK prevents all steps of the fibrogenesis process, since the inflammatory damage, the fibroblast proliferation, and the excessive ECM synthesis [89]. Similarly, to AMPK, the growth differentiation factor 11 (GDF11), a member of the transforming growth factor-β superfamily, exhibits less expression levels with aging [91,92]. In humans, lower levels of GDF11 have been linked to left ventricular hypertrophy [93] and restoration of GDF11 levels reverses age-related cardiac hypertrophy in mice [91,92].

### 2.2 Amyloidosis

Amyloidosis is a clinical disorder caused by unstable protein structures that misfold, deposit and form amyloid fibrils, causing cellular, tissue and organ disfunction [94,95]. It has been reported two types of transthyretin (TTR) amyloidosis in cardiac tissue of aged individuals: wild-type transthyretin (wtTTR), also known as senile cardiac amyloidosis and mutant or hereditary amyloidosis (mTTR) [96]. Both forms, wtTTR and mTTR, cause restrictive cardiomyopathy, with thickened and stiffened ventricles that leads to diastolic and systolic dysfunction, arrhythmias, and clinical heart failure [96]. The wtTTR cardiac amyloidosis is the most prevalent form that affects mainly males over the age of 60 and represents a common cause of diastolic heart failure in elderly population [97]. Interestingly, approximately 25% of autopsied hearts from individual over 80 years, present wTTR amyloid deposits [98]. The clinical features of cardiac wtTTR amyloidosis did not distinguish from the other types [99]. Usually, patients present dyspnea on exertion (86%), peripheral edema (64%), and atrial fibrillation (67%) with a ranging onset from 60 years to almost 88 years of age. Nonetheless, the prevalence of atrial fibrillation is higher in wtTTR amyloidosis patients when compared between age-matched patients with other types of amyloidosis, where occurrence of atrial fibrillation is almost equal to general elderly population [97,100]. The molecular mechanisms underlining senile cardiac amyloidosis are not well understood, but age-related events such as post-translational biochemical modifications in wtTTR or its chaperones seem to contribute to form amyloid fibrils. Studies from mice overexpressing human wtTTR suggest that alterations in the production of hepatic chaperone or proteasome clearance of unfolded protein determine the depositions of amyloidosis in the aging heart [100].

### 2.3 Mitochondrial dysfunction

The mitochondria play a crucial role regulating a myriad of cellular processes including oxygen sensing, ATP production, cellular redox homeostasis, and regulation of programmed cell death [101]. The maintenance of the cardiac function requires a vast amount of energy where mitochondria are critical to produce ATP in the myocardium. Thus, age-associated mitochondrial dysfunction results in important physiological cellular impairments that contributes to the development of cardiovascular diseases [102,103]. Cardiac aging is accompanied by a decline in the mitochondrial function, which leads to increase the productions of reactive oxygen species (ROS) due to mitochondrial oxidative phosphorylation. An increment of ROS, including superoxide [O_2_^-^] and hydrogen peroxide [H_2_O_2_], drives to cellular oxidative stress, one of the most important phenomena associated to age-related mitochondrial dysfunction [102,104,105]. Besides mitochondrial oxidative phosphorylation, ROS production has other sources such as NADPH oxidase at the plasma membrane, lipid oxidation within peroxisomes and various cyclooxygenases and xanthine oxidase in the cytoplasm [102,104]. Accumulation of mitochondrial ROS cause damage to several components of the mitochondria, including mitochondrial DNA mutations and oxidation of respiratory enzymes. This last, will increase mitochondrial production of ROS and therefore, further mitochondrial damage [102]. In fact, mitochondria are particularly vulnerable to oxidation due to the lack, in the mitochondrial DNA (mtDNA), of protective histones [104]. In a mouse model of mice carrying defective mitochondrial DNA polymerase gamma (PoIG), has been demonstrated that mice present significant mtDNA deletions [106]. This mouse model exhibits global features of premature aging including cardiac hypertrophy, fibrosis, and systolic and diastolic dysfunction. In additional, PoIG mutant mice presents a reduction on the mitochondrial biogenesis, respiratory chain impairments and increased cell apoptosis [107,108]. In mice that overexpressed catalase targeted to the mitochondria (mCAT), have increased the lifespan around 20% when compared with WT littermates [109,110]. Further, mCAT mutants present a delay in the development of cardiac-specific impairments, a reduction of oxidative damage, along with an attenuation of H_2_O_2_ production and H_2_O_2_-induced aconitase inactivation [109]. In addition, mCAT mice display an improvement of LV mass index, diastolic and systolic function, and myocardial performance index when compared with age-matched WT. The mitochondrial damage in aged hearts stimulates an increase of mtDNA copies along with upregulation of transcription factors involved in mitochondrial biogenesis, such as the master regulator PPAR-γ Coactivator-1-α (PGC-1α). These changes were found to decreased in mCAT mice, further supporting the role of mitochondrial ROS in cardiac aging [110].

Additional implication of mitochondria in cardiac aging arrives from studies on the mitochondrial adaptor *p66^Shc^*. The *p66^Shc^* localizes to the mitochondrial intermembranous space and plays a pivotal role in ROS generation and cellular apoptosis [111]. The *p66^Shc^* is phosphorylated by PKA-β and prolyl isomerase Pin-1. The phosphorylated isoform of *p66^Shc^* can accumulate in the mitochondria where it activates mitochondrial Ca^2+^ response, and then induce cellular apoptosis [112,113]. In cells where *p66^Shc^* gene was abolished, presents a significant reduction of intracellular free radicals. Likewise, mice with a targeted mutation of the *p66^Shc^* gene, exposed to high oxidative stress display less global ROS [114]. Oxidative stress is enhanced with diabetes and prone to the onset of cardiac modifications. Genetic deletion of *p66^Shc^* has been demonstrated to prevent angiotensin-II-induced LV hypertrophy and cardiomyocytes apoptosis in a diabetic mouse model. It was also observed a significant reduction of oxidative damage in cardiac progenitor cells, cardiomyocytes, and endothelial cells [115].

Typically, there is a trend to link mitochondrial dysfunction to increased generation of ROS, however, PoIG mutants have minor or no significant increase of oxidative stress [107,116]. Thus, ROS-independent mechanisms seem to be involved in premature aging. These mechanisms may involve metabolic mitochondrial damage in critical subunits or when a phenotypic threshold effect occurs. In human aged cardiomyocytes it has been found the presence of large fractions of mtDNA mutations suggesting that such mutations play a role in the cardiac aging process [117]. The mitochondrial transcription factor A (Tfam) functions as a regulator of transcription and replication of mtDNA. In a mouse model that lacks Tfam, animals develop progressive respiratory chain deficiency, dilated cardiomyopathy, atrioventricular blockage and end up dying at 2-4 weeks of age [118]. Likewise, mitochondrial dysfunction is observed in mice expressing a dominant-negative mutant mitochondrial helicase. These mutation leads to the accumulation of mtDNA deletions and thus, mice develop a mosaic cardiac-specific progressive respiratory chain deficiency, spontaneous ventricular premature contractions, and arrhythmias, that can be consequence of abnormal Ca^2+^ [119].

### 2.4 Calcium signaling impairments

The ion Ca^2+^ plays a crucial role in the excitation-contraction coupling (ECC). The ECC is initiated by an action potential that drives cardiomyocytes membrane (sarcolemma) excitation. The depolarization of the sarcolemma leads to a fast opening of voltage-gated Na^+^ channels and further membrane depolarization that allows the opening of voltage-gated Ca^2+^ channels (mainly L-Type Ca^2+^ channels). The Ca^2+^ influx triggers a process termed Ca^2+^-induce Ca^2+^-release via the opening of ryanodine receptor (RyR) in the sarcoplasmic reticulum (SR). The Ca^2+^ coming from the SR binds to troponin-C of the troponin-tropomyosin complex on the actin filaments of the sarcomeres leading to cardiomyocytes contraction [120]. The repolarization of the sarcolemma is mediated by voltage-gated K^+^ channels opening that generates outward currents and the action potential repolarization. In this process, the Ca^2+^ dissociates from troponin-C and is taken back up into the SR via phospholamban (PLB)-regulated Ca^2+^-ATPase (SERCA2a) and is removed from the cytosol through the sarcolemma Na+/Ca^2+^ exchanger (NCX) [120].

In aged cardiomyocytes, impaired Ca^2+^ handling results from age-related modification in proteins associated with the ECC [121-123]. The expression levels of SERCA2a decreased with aging and seems to be the primary mechanisms for the extended Ca^2+^ transients [124]. Furthermore, it has been reported that diminished SERCA2a levels play a main role in the development of diastolic dysfunction in elderly. The cardiac overexpression of SERCA2a in senescent rats, improved myocardial contractility and diastolic function [125]. In addition, alteration in SERCA2a regulatory proteins has been documented. Studies in old and senescent mice hearts, demonstrated a significant reduction of SERCA2a/PLB and NCX ratios [126]. The ẞ-adrenergic-mediated cAMP accumulation and phosphorylation of PLB and troponin I is found to be diminished in aging rat heart. Thus, a decreased cAMP/PKA/PLB activity leads to an impaired contractile response of the aged heart to β-adrenergic stimulation [127]. Finally, the Ca^2+^/calmodulin-dependent protein kinase II (delta-isoform) has found to be nearly 50% lower in the aged rat hearts, which contributes to impaired SR function [113,128].

## 3. Genetics and epigenetics in the developments of cardiovascular diseases

### 3.1 DNA mutations in cardiovascular aging

During the aging process the accumulation of genetic and epigenetic changes contributes to the development of cardiovascular diseases [129]. The genetic damage can be divided in three major categories: chemical DNA damage, mutations that can occur in parts of the genetic code and epigenetic modifications that lead to changes in the gene activity without affecting the DNA sequence [130,131]. Even though systems exhibit efficient mechanisms to repair DNA, DNA damage occurs and largely contribute to cellular and organ dysfunction in the cardiovascular aging process [132]. Genetic modified mouse models with severe genomic instability due to defective nucleotide excision repair genes *ERCC1* and *XPD* recapitulate age-dependent vascular dysfunction, endothelial cells senescence and elevated blood pressure at a very young age. The age-dependent cardiovascular dysfunction, in this mouse model, seem to be related to a decrease of endothelial nitric oxide (eNO) synthase levels, sirtuin deregulation, increased levels of NADPH oxidase [133] and impaired phosphodiesterase (PDE) activity [133,134]. Likewise, Human vascular smooth muscle cells have augmented the mRNA levels of PDE1A, PDE1C and PDE5, which is related with markers of cellular senescence. Moreover, the PDE1A single nucleotide polymorphisms have been demonstrated to be linked to impaired diastolic blood pressure and carotid intima-media thickness [134].

Mutations in the *LMNA* gene (encodes lamin A and C, structural protein components of the nuclear lamina) cause Hutchinson-Gilford progeria syndrome, a genetic condition characterized by the dramatic and rapid appearance of aging beginning in childhood. Further, individuals with this syndrome develop premature arteriosclerotic disease and cardiovascular dysfunction that leads to cardiac attacks and strokes at a mean age of 13 years [135].

Somatic DNA mutations have been observed in circulating cells of patients with atherosclerosis and in the plaques [136]. Individuals with coronary heart disease have chromosomal damage and defective mitochondrial DNA in blood mononuclear cells. The consequences of DNA damage, including cell senescence, apoptosis, or growth arrest, in plaques correlate with disease severity [136]. Moreover, the genetic variations in the nucleotide excision repair components (rs2029298) in the putative promoter region of the *DDB2* gene are tightly associated with carotid femoral pulse wave velocity [133]. Finally, several studies have demonstrated that genomic damage and the accumulation of somatic mutations accompanies cardiovascular aging and are associated with development of cardiovascular disease [137].

### 3.3 Chromatin modifications in cardiovascular aging

Epigenetic modifications caused by the environmental interactions on the genome to alter gene expression can be durable, stable and inherited. These modifications can act on several transcriptional programs (involved in the cellular metabolism, oxidative stress, and inflammation) disrupting them [138]. Thus, it seems reasonable that accumulation of epigenetic changes in the aging process contribute to the appearance of early cellular senescent and associated cardiovascular disease [102,139-141]. Chromatin changes can be grouped in two main categories: DNA methylations and histone modifications. DNA methylations involve changes in the nucleotides that receive a methyl group (in the 5′ cytosine of C-G dinucleotides, referred as CpGs). Consequently, the addition of the methyl group in the CpG nucleotides affects the chromatin accessibility to the transcriptional machinery and thus, increase or represses the gene transcription [142].

Histone acetylation and deacetylation are important contributors to aging and age-related diseases, including atherosclerosis. In fact, in mice models has been demonstrated that changes in methylation occur before the appearance of the first symptoms. Moreover, atherogenic lipoproteins plays a role in the DNA methylation and histone post-translational modifications in the human monocyte cells [143]. Increased hypermethylation and histone acetylation rise the expression levels of the adaptor protein p66Shc in aging and, its availability for activation [144].

Chronic stress, one of the major environmental factor that drives epigenetic modifications, has been documented to affect cardiovascular system. In fact, chronic stress is associated with a decreased in DNA methylation [145]. Impaired patterns of DNA methylation and DNA acetylation of genes encoding matrix metalloproteinases are linked to the development of aortic aneurism [146]. The histone deacetylase expression is increased in Human and mice with abdominal aortic aneurism and the treatment with class I and IIa histone deacetylase inhibitors limit aneurysm expansion in mice [147]. In addition to the development of aortic aneurisms, impaired methylation of the cardiac DNA due to mitochondrial cardiac polymerase dysfunction, contributes to the appearance of cardiomyopathy in young and old rats. Nevertheless, cardiac DNA methylation present different pattern between young and aging rats being more robust in these aged heart [148].

### 3.4 Telomeres shortening and cellular senescence

The telomers consist of several repetitions of nucleotide sequences (TTAGGG) in the extreme end of the mammalian chromosomes. Telomers has the function to preserve the stability and integrity of the chromosomes from deterioration or preventing the fusion with neighboring chromosomes [149]. Telomers shortening has been linked to cellular senescence, a phenomenon that significantly contributes to structural and functional modification of the aging heart. With each cell division the telomeric DNA shortens till reach a critical length. Once reaches this point, the chromosomal ends are exposed to damage which leads to cell senescence, and finally apoptosis. Telomere shortening has relevance in the context of age-related cardiovascular diseases [150-153]. Moreover, there is a positive association of decreased leukocyte telomere length (LTL) with increased risk of stroke, myocardial infarction, type 2 diabetes mellitus, diastolic blood pressure, aortic valve stenosis and vascular cell senescence [150,154]. In a nested case-control study has been reported that patients with clinical and subclinical symptoms of atherosclerosis exhibit short LTL when compared with healthy individual [155]. In another case-control study, patients with shorter LTL length had a higher occurrence of atherothrombotic stroke or hemorrhagic stroke when compared the lowest to highest tertile of telomere length [156]. In addition, individual in the lowest LTL tertile had a marked augmented risk for incident plaque and plaque progression [157]. Finally, it has been demonstrated that shortest LTL are associated with higher relative risk for coronary heart disease and in a lesser extend for cerebrovascular [158].

Telomerase KO mice (TERC^-/-^) exhibit a progressive shortening telomere in each generation. These telomeres shortening is accompanied by an attenuation in the cardiomyocyte’s proliferation and an increase of cell apoptosis [159]. Further, TERC^-/-^ mice have a thicker ventricular wall and ventricular dilatation that results in ventricular dysfunction which mimics human dilated cardiomyopathy observed in the end-stage. In the other hand, an overexpression of the telomerase in cardiomyocytes increases the cell proliferation and leads to cardiac hypertrophy [160]. In insulin growth factor-1 transgenic mice the enhanced telomerase activity prevents ventricular dysfunction which seems to be related with a reduction of cellular aging and an increase of cell growth [161]. The telomeres shortening is also related to the development of heart failure. Endometrial biopsies from patients with heart failure have demonstrated a significant reduction of telomeres length and an associated increase of myocytes senescence and cell death [162]. Thus, it is suggested that telomeres play an important role in the age-related cardiomyopathy and in the pathophysiology of heart failure [163,164].

## 4. Aging heart and response to the exercise

The exercise requires a fast adaptation of the body that involves a complex and coordinated response of the cardiovascular, pulmonary, and nervous system [165]. It is known that at rest, the muscles receive approximately 20% of the total blood flow, whereas during exercise, the demand of blood can increase above 80% [166].The aging process leads to changes in these systems disrupting its normal function. Along with aging and due to the impairments in the communication between systems is observed a significant decrease in peak cardiac output (CO) and in the capacity response to exercise [167]. In response to exercise, the CO augments to supply the enhanced cellular metabolic demands. This physiological adaptive response to exercise depends on two physiological parameters, the heart rate (HR) and stroke volume (SV). Both heart rate and stroke volume rapidly increase due to exercise-induced adrenergic stimulation. The SV augments as result of augmented myocardial contractility and decreased peripheral resistance. In response to exercise, the SV increases in a proportional way of exercise intensity. The SV may increase 40-50% of maximal capacity tending to reach then a plateau. Then, and additional increment of CO is achieved by further increase of HR [168]. With aging, the capacity to augment CO in response to exercise is affected. Thus, in aging, the maximal HR decreases, taking place a chronotropic incompetence, which constitutes the main contributor of the reduced cardiac response to exercise. It has been reported that in normal aging, the maximal HR progressively decrease around 0.7 beats per minute each year [169]. Chronotropic incompetence is considered an important cause of exercise intolerance, but also an independent predictor of cardiovascular events and mortality. One third of patients with HF present chronotropic incompetence and contribute for the maladaptive symptoms in response to exercise. The mechanisms underlying chronotropic incompetence are not well stablished, however, it has been suggested that a reduced β-adrenergic density and/or sensitivity and impaired autonomic regulation may play a central role [165,170].

The effects of cardiac aging on the elevation of SV with exercise are not well established. Overall, in aged hearts SV are still augmenting in response to exercise, it has been noted that this increase is not enough to compensate for the reduction of the maximum HR. In adult young hearts myocardial contraction is the major responsible for the increase of SV. However, in aged heart, the exercise-associated increased of SV is mainly due to the increase of end-diastolic volume [171].

Advanced aging is also accompanied by a decline in the maximal aerobic capacity (MAC) along with modifications in the mechanisms that regulate oxygen dynamics. The age-linked decline in the MAC increases gradually and is estimated to decrease each decade of life. Studies reveal that MAC decreases 3-6% during the fourth decades of live and more than 20% after reaching age 70 [172,173]. The drop in the aerobic capacity can be attributed to alteration in the regulation of the oxygen dynamics in response to exercise [172,174,175]. The insufficient oxygen delivery from impaired cardiac reserve (incapacity of the heart to effectively augment CO) represents a major contributor to diminished functional capacity in aging, particularly in those patients with HF [175,176]. In normal aging, the maximum oxygen consumption, a metric of exercise capacity, decrease around 10% per decade of live, and this decline considerably accelerates in individual after 70 years and especially in patients with HF [172]. The age-related decline of CO and arteriovenous oxygen difference in response to excise is consistent with the marked reduction observed in the mitochondrial function, mitochondrial enzymes activity and capillary density [102], suggesting that impairments in the cellular respiration and peripheral mechanisms of oxygen extraction plays a role in the effects of aging on the cardiac response to exercise [8,165].

## 5. Conclusions

**Figure 4.**
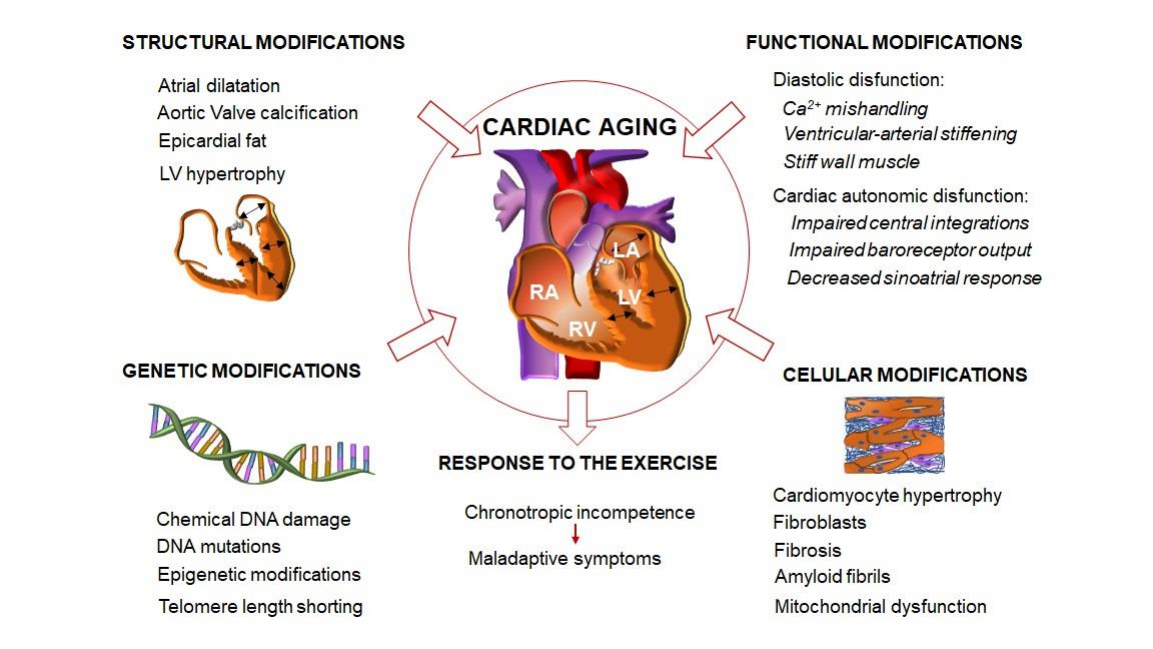
**Graphic summary of the aging heart**. Major cardiac structural modifications: left atrium dilatation caused by the increase of diastolic pressure because of slower ventricular filling; cardiac hypertrophy, characterized by a significant increase of the LV wall with a reduction of the LV chamber size; Aortic valve calcification and epicardial fat accumulation. Major functional modifications: diastolic function declines due to impairments of Ca^2+^ cycling/handling, affected by oxidative damage of SERCA, and ventricular-atrial stiffening in the senescence myocardium; systolic function is usually preserved at least at rest. Cardiac autonomic dysfunction due to impaired central integrations, impaired baroreceptor output and decreased sinoatrial response. Major cellular modifications: total number of cardiomyocytes declines, and the remaining undergo hypertrophic; fibroblast cells proliferation; increase fibrosis and amyloid fibrils formation; mitochondrial dysfunction that leads to ROS accumulation. ROS drives cellular oxidative stress and mtDNA damage which is reflected in an impairment of the energetic and metabolic state that disrupts homeostasis. Major genetic modifications include chemical DNA damage, mutations in the DNA and epigenetic modifications that change genes activity; telomere length shortening that leads to cell senescence and apoptosis. Cardiac adaptation to exercise: maximal HR decreases leading to chronotropic incompetence and thus to maladaptive symptoms in response to exercise. To compensate for the reduction of maximal HR, SV increases, however it seems to be slightly affected in the aged heart. Moreover, the MAC decreases which contributes to modifications in the mechanism that regulates oxygen dynamics in response to exercise.

The evidence we collected in this literature review suggests that aging is the major risk factor for the development of cardiac disease conduction to heart failure and mortality. As summarized in Figure 4, the major age-related structural changes occur in the left side of the heart: cardiac hypertrophy, characterized by a significant increase of the LV wall with a reduction of the LV chamber size. The increase of the LV mass is more pronounced when associated with CV risk factors such as diabetes or hypertension; on the other hand, the left atrium undergoes a progressive increase in the diameter of the chamber caused by the increase of diastolic pressure because of slower ventricular filling.

Diastolic function declines with aging whereas systolic function is likely to be preserved at least at rest. The leading cause of diastolic function is related to impairments on the Ca^2+^ cycling/handling, affected by the oxidative damage of the SERCA, and due to the ventricular-atrial stiffening in the senescence myocardium.

At cellular levels there is an increase of fibrosis and amyloid fibrils formation. Even though the molecular mechanisms promoting cardiovascular fibrosis are not well understood, cytokines, angiotensin II, AMPK, TGF-β and Gal-3 seem to play an important role in the development of age-related cardiac fibrosis. On the other hand, TTR amyloidosis increases with aging. Both isoforms lead to restrictive cardiomyopathy, diastolic and systolic dysfunction, arrhythmias, and heart failure.

Mitochondrial dysfunction that occurs with aging, leads to ROS accumulation. ROS drives cellular oxidative stress and mtDNA damage which is reflected in an impairment of the energetic and metabolic state that disrupts homeostasis.

Genetic modifications occur during the aging process and eventually lead to the development of age-related cardiovascular diseases. These genetic modifications can be categorized in 3 groups including chemical DNA damage, mutations in the DNA and epigenetic modifications that change genes activity.

Telomere length shortens with age and leads to senescence and apoptosis and are tightly related with the development of cardiovascular diseases and heart failure. In aged humans and animal models of aging, telomere shortening is observed and is linked to higher risk of coronary heart disease, atherothrombotic or hemorrhagic stroke.

Cardiac adaptation to exercise is affected by aging. The maximal HR decreases leading to a chronotropic incompetence. Chronotropic incompetence contributes to the maladaptive symptoms in response to exercise. To compensate for the reduction of maximal HR, SV increases, however it seems to be slightly affected in the aged heart. Moreover, the MAC decreases which contributes to modifications in the mechanism that regulates oxygen dynamics in response to exercise.
